# From Policy to Practice: Brazil's Pathway to Eliminating Vertical Transmission of HIV as a Public Health Milestone

**DOI:** 10.1002/jia2.70123

**Published:** 2026-06-19

**Authors:** Pâmela Cristina Gaspar, Angélica Espinosa Miranda, Leonor Henriette de Lannoy, Ana Roberta Pati Pascon, Carmen Silvia Bruniera Domingues, Ítalo Vinicius Albuquerque Diniz, Amanda Krummenauer, Ana Paula Betaressi da Silva, Alisson Bigolin, Ariane Tiago Bernardo de Matos, Márcia Rejane Colombo, Claudia Gonçalves Siqueira, Angela Gasperin Martinazzo, Adele Schwartz Benzaken, Draurio Barreira, Mariângela Batista Galvão Simão, Brazilian Elimination Vertical Transmission Group (Brazilian EVT Group), Adria Albarado, Adson Belém Ferreira da Paixão, Alana Souza de Araujo, Alessandro Ricardo Caruso da Cunha, Amanda Alencar Cabral Morais, Ana Cláudia Philippus, Antônio Ramos de Carvalho, Aparecida Morais Lima, Artur Olhovetchi Kalichman, Camila Márcia Mendes, Cristiano Francisco da Silva, Cristina Alves Pessoa Candido, Fabio Moherdaui, Fernanda Lopes Conte, Francisca Lidiane Sampaio de Freitas, Graziela de Queiroz Macedo, Iêda Maria Oliveira Fornazier, Isabella Mayara Cleide Diana de Souza, Ivo Brito, Jair Brandão Moura Filho, José Boullosa Alonso Neto, Juliana Santos Moreno, Katherine Jeronimo Lima, Lilian Nobre de Moura, Leila Suely Araujo Barreto, Léssio Antônio Nascimento Júnior, Luiz Fernando Aires Júnior, Luzia Pereira de Lima, Matheus Funke Spinelli, Mayra Gonçalves Aragón, Moyra Machado Portilho, Nádia Maria da Silva Machado, Patrícia Werlang, Rafael Chitolina, Rayone Moreira Costa Veloso Souto, Rogger Diquique, Romina do Socorro Marques de Oliveira, Ronaldo de Almeida Coelho, Sandra Regina de Souza Lobato Miguel, Sarah Maria Soares Fernandes Bayma, Silvana Pereira Giozza, Vinicius da Motta de Mello

**Affiliations:** ^1^ Department of HIV, AIDS, Tuberculosis, Viral Hepatitis, and Sexually Transmitted Infections, Secretariat for Health and Environmental Surveillance Ministry of Health of Brazil Brasília Brazil; ^2^ Postgraduate Program in Infectious Diseases Health Sciences Center Federal University of Espírito Santo Vitória Brazil; ^3^ Heitor Vieira Dourado Tropical Medicine Foundation, State University of Amazonas (UEA) Manaus Brazil; ^4^ Secretariat for Health and Environmental Surveillance Ministry of Health of Brazil Brasília Brazil

**Keywords:** certification, community engagement, elimination, health plan implementation, HIV, infectious disease transmission, universal healthcare, vertical

## Abstract

**Introduction:**

The elimination of vertical transmission of HIV is a global health priority endorsed by the Pan American Health Organization (PAHO) and the World Health Organization (WHO), aligned with the Sustainable Development Goals (SDGs). Brazil has made substantial progress towards this goal through sustained investment in universal health policies and a rights‐based approach to healthcare.

**Discussion:**

Brazil's national HIV response is anchored in the Unified Health System (Sistema Único de Saúde—SUS), a universal, decentralized and participatory health system that ensures access to prevention, diagnosis, treatment and monitoring. Tripartite governance—across federal, state and municipal levels—combined with strong community engagement, has enabled coordinated policy implementation at scale. This framework has supported high coverage of antenatal care (>98%), HIV testing (>95%) and antiretroviral therapy among pregnant women living with HIV (>95%) in recent years. Vertical transmission rates of HIV declined from 3.73% in 2015 to 1.78% in 2023, remaining below the elimination threshold. The annual rate of new paediatric HIV acquisitions also declined to low levels, being 5.99 per 100,000 live births in 2023, far below the PAHO/WHO targets. A certification for states and municipalities strategy further accelerated progress toward the elimination by strengthening accountability, improving data quality and engaging local health systems.

**Conclusions:**

Brazil's experience demonstrates that HIV vertical transmission elimination is feasible in large and unequal settings when supported by strong governance, integrated health systems and sustained political commitment. The certification of vertical transmission elimination of HIV by PAHO/WHO in 2025 represents a major milestone and offers valuable lessons for global health. Brazil remains committed to sustaining stainability of this certification and to advancing efforts for the elimination of other vertically transmitted infections, including syphilis, hepatitis B, Human T‐cell lymphotropic virus (HTLV) and Chagas disease.

## 1 Introduction

The elimination of vertical transmission of HIV is a major global health priority endorsed by the Pan American Health Organization (PAHO) and the World Health Organization (WHO), aligned with the Sustainable Development Goals (SDGs) [1, 2, 3]. Achieving this target requires sustained political commitment, robust healthcare systems and equitable access to prevention, diagnosis, and treatment services.

Since the establishment of the National AIDS Program in 1985 [4], Brazil has made substantial progress towards eliminating vertical transmission of HIV as a public health problem. Over the past four decades, the country has developed a comprehensive and sustained national response grounded in universal health coverage, social participation and rights‐based policies, supported by coordinated commitments across federal, state and municipal levels. These commitments include the Health Pact (2006) [5, 6], the National Pact for the Elimination of Vertical Transmission of HIV, Syphilis, Hepatitis B and Chagas Disease (2022) [7], and the Healthy Brazil Program—Uniting to Care (Programa Brasil Saudável—Unir para Cuidar), which adopts an intersectoral approach to eliminate, by 2030, diseases shaped by social determinants, including those transmitted vertically [8, 9]. This trajectory led to Brazil's submission of its report to PAHO/WHO for validation of the elimination of vertical transmission of HIV [10].

This commentary describes Brazil's pathway to achieving certification of the elimination of vertical transmission of HIV as a public health problem in December 2025. It examines governance, health system organization, nationally coordinated certification strategies for states and municipalities, and epidemiological outcomes, and discusses ongoing challenges and lessons relevant to global health policy and implementation.

## 2 Discussion

### 2.1 Governance and Health System Foundations

Brazil's progress in reducing vertical transmission of HIV is closely linked to the institutional architecture of its Unified Health System (Sistema Único de Saúde—SUS), established by the 1988 Federal Constitution and regulated by subsequent legislation. The SUS is grounded in the principles of universality, comprehensiveness and equity, ensuring free and universal access to health services. Beyond its normative framework, the SUS operates as a policy delivery platform that enables large‐scale implementation of public health interventions in a highly heterogeneous context [11, 12].

The governance of the SUS is decentralized and participatory, involving shared responsibilities across federal, state and municipal levels. This tripartite model enables coordinated policy implementation while allowing adaptation to local contexts. Participatory governance is reinforced by institutional mechanisms such as the national health council, which include representatives from civil society, health professionals and government. The Council plays a central role in the formulation, monitoring and oversight of the National Health Policy, including its economic and financial dimensions, and in promoting social control across both the public and private health sectors [11, 12].

Since the early years of the HIV epidemic, Brazil's response has been shaped by strong engagement from civil society and social movements. This collaboration has been instrumental in promoting rights‐based approaches, reducing stigma and ensuring universal access to antiretroviral therapy (ART), which has been provided free of charge and guaranteed as a legal right since 1996 [13]. The integration of governance, social participation and sustained public investment has created a resilient policy environment that supports the implementation of comprehensive HIV prevention and care strategies, including those targeting vertical transmission.

### 2.2 Integrated Strategies and Health System Capacity

Brazil's approach to vertical transmission elimination is embedded within a broader strategy that integrates HIV, syphilis, hepatitis B, Human T‐cell lymphotropic virus (HTLV) and other vertically transmitted infections into maternal and child health services [14]. This integrated model ensures continuity of care throughout pregnancy, delivery and postpartum, while requiring effective integration between primary healthcare and specialized services to support timely diagnosis and appropriate clinical management, and to address social and structural determinants of health. Key interventions include universal antenatal care, routine HIV testing, immediate initiation of ART for pregnant women living with HIV and/or AIDS, and appropriate management of HIV‐exposed infants, in accordance with national clinical protocols [14, 15]. These actions are supported by centralized procurement of essential supplies, including antiretrovirals, diagnostic and monitoring tests, and prevention technologies.

A critical strength of Brazil's response lies in the integration and linkage of multiple national health information systems, including surveillance, laboratory, treatment and mortality databases. This integrated data infrastructure enables near real‐time monitoring of the HIV care continuum and supports validation of elimination indicators at both national and states and municipalities levels [10]. Importantly, these systems function not only as monitoring tools but also as instruments of governance, enabling continuous performance assessment and evidence‐informed policy adjustments.

Estimates of ART coverage among pregnant women living with HIV, vertical transmission rates and paediatric HIV incidence were derived through the linkage of multiple national health information systems, including SINAN (case notifications), SICLOM (ART dispensation), GAL (laboratory results, including proviral DNA), SISCEL (viral load and CD4 counts) and SIM (mortality data). Data quality was further strengthened through active case verification of HIV‐exposed children, based on viral load records, in collaboration with state, municipalities and local health services to confirm cases and identify gaps in follow‐up. Additionally, antenatal care coverage and population denominators were obtained from SINASC, Brazil's national live birth system. This integrated data ecosystem supports comprehensive monitoring and validation of vertical transmission elimination indicators at national, state and municipal levels [10, 16].

The assessment of indicators underpinning the certification was conducted using consolidated data from national HIV information systems covering both public and private healthcare sectors, enabling validation of most certification indicators. HIV testing coverage among pregnant women was estimated through a sample‐based methodology designed by the Ministry of Health to ensure national representativeness, including all state capitals and municipalities with more than 100 live births per year. A total of 50 municipalities and 1715 pregnant women per year (2023–2024) were included to assess antenatal HIV testing coverage [10].

Also, the Ministry of Health, in partnership with states and municipalities, maintains a nationwide laboratory network for HIV diagnosis and monitoring, ensuring access to viral load testing and early infant diagnosis. This network combines centralized high‐capacity laboratories with decentralized near point‐of‐care services, enabling broad coverage across diverse geographic settings. Additional specialized tests are provided through national reference laboratories, while routine diagnostics are widely available across all federative units. A national External Quality Assessment programme further ensures the reliability and standardization of rapid and laboratory testing [10].

### 2.3 Certification for States and Municipalities Driving Nationwide Elimination of Vertical Transmission

Recognizing its geographic and socioeconomic heterogeneity, Brazil adapted the PAHO/WHO framework for the elimination of vertical transmission [1, 2] to include a certification strategy for states and municipalities [17, 18]. This approach allows states and municipalities with populations of ≥100,000 inhabitants to be evaluated and recognized by the Ministry of Health based on their performance in achieving elimination targets. The certification process includes the assessment of impact and process indicators, as well as qualitative dimensions such as governance, data quality, service organization, respect for human rights and community engagement. States and municipalities can be certified for elimination or awarded progressive tiers of good practices, encouraging continuous improvement.

The certification process for states and municipalities for the elimination of vertical transmission of HIV in Brazil began in 2017 and has progressively expanded in scope and scale. Initially focused on HIV, the strategy was later extended to include syphilis, along with the introduction of progressive tiers of good practices, enabling continuous improvement even in settings not yet eligible for full elimination. Subsequently, the framework was expanded to incorporate additional infections, including hepatitis B, Chagas disease and HTLV, reflecting a comprehensive approach to vertical transmission [17, 18]. By 2025, a substantial number of municipalities and several states had achieved certification or recognition for good practices, and territories may hold multiple certifications across different infections.

Eligibility for certification includes municipalities with ≥100,000 inhabitants and all states, which may apply either for full elimination or for progressive tiers of good practices (bronze, silver and gold) designed to support gradual reductions in vertical transmission rates. Applications are assessed through documentation review and validation missions conducted by the National Validation Team (NVT). The NVT also provides feedback to local managers, including tailored recommendations to strengthen healthcare delivery and surveillance practices. In turn, the NVT identifies best practices in the field and promotes their dissemination across territories, thereby improving related processes. The NVT prepares a report that is reviewed and discussed by the National Validation Committee, which makes the final decision and recommendations [17, 18].

In this context, the certification process operates as a performance‐based governance mechanism, reinforcing accountability and promoting continuous quality improvement. By linking recognition to measurable indicators and qualitative assessments, the strategy contributes to the development of a learning health system, in which local experiences inform national policy and best practices are disseminated across jurisdictions.

### 2.4 Epidemiological Progress Towards Elimination

**FIGURE 1:**
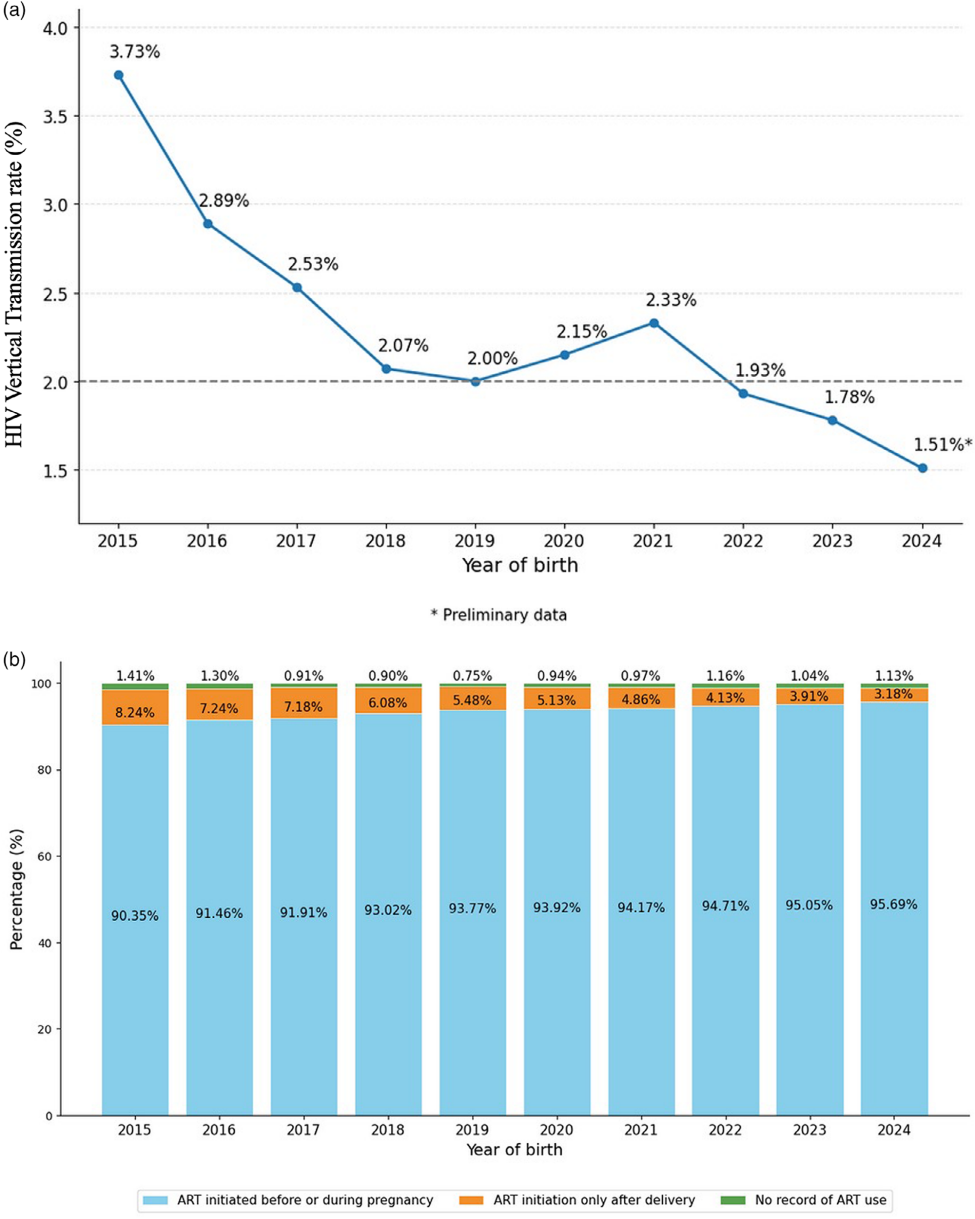
(A) HIV vertical transmission rates in Brazil, 2015–2024. *Source*: Elaborated by the authors, based on the Brazilian Validation Report for the Elimination of vertical transmission of HIV as a public health problem [10]. (B) ART initiation among pregnant women living with HIV by year of birth, Brazil, 2015–2024. Abbreviation: ART, antiretroviral therapy. *Source*: Elaborated by the authors, based on the Brazilian Validation Report for the Elimination of vertical transmission of HIV as a public health problem [10].

Brazil has achieved substantial reductions in vertical transmission of HIV over the past decade. The vertical transmission rate declined from 3.73% in 2015 to 1.78% in 2023, remaining consistently below the 2% threshold established by PAHO/WHO [10, 16]. As shown in Figure 1A, this reflects a sustained downward trend over time, with stabilization at low levels in recent years. This progress is supported by high coverage of key interventions. Antenatal care coverage exceeds 98%, and HIV testing among pregnant women surpasses 95%. Among pregnant women living with HIV, ART coverage also increases across the years and exceeds 95% (Figure 1B), reflecting effective linkage to care and adherence to treatment protocols [10, 16]. The annual rate of new paediatric HIV acquisitions has declined to 5.99 cases per 100,000 live births in 2023 and remains well below global elimination targets [10, 16].

### 2.5 Persistent Challenges and Inequalities

Despite substantial progress, persistent regional and social inequalities remain a critical challenge to sustaining the elimination of vertical transmission. Brazil's continental dimensions and heterogeneous distribution of health infrastructure result in unequal access to timely diagnosis, treatment and follow‐up care. These disparities are particularly pronounced in remote regions such as the Amazon and among Indigenous and other vulnerable populations [19, 20].

Structural determinants—including poverty, stigma, gender inequality and educational barriers—interact with health system limitations, affecting both access to services and continuity of care [19, 20, 21, 22]. These inequities pose a risk not only to achieving but also to sustaining elimination targets over time. These disparities highlight that sustaining elimination requires not only maintaining coverage but also addressing structural inequities that affect access and continuity of care.

To mitigate these challenges, Brazil has expanded primary healthcare coverage through the Family Health Strategy and implemented targeted interventions for vulnerable populations [23]. However, sustained efforts are required to ensure equitable access and maintain progress across all regions.

### 2.6 Implications for Global Health

Brazil's experience offers important lessons for countries seeking to eliminate vertical transmission of HIV, particularly those with large populations and marked inequalities. The combination of universal health coverage, decentralized governance, strong community engagement and integrated health information systems provides a robust foundation for implementation.

Whereas early successes in vertical transmission elimination were largely observed in smaller and more homogeneous settings, Brazil demonstrates that elimination is feasible within a large, decentralized health system operating across highly heterogeneous contexts. Its certification strategy for states and municipalities represents an innovative approach to operationalizing global targets in complex settings and may inform strategies to advance elimination in similarly complex contexts.

## 3 Conclusions

The elimination of vertical transmission of HIV as a public health problem in Brazil, certified in December 2025 by PAHO/Who, reflects more than four decades of sustained investment in the HIV response, embedded within universal health policies grounded in equity, participatory governance and strong civil society engagement. Broad access to prevention, testing and treatment, together with consistent adherence to clinical guidelines and robust national epidemiological surveillance systems, underscores the strength of this response. Brazil's Unified Health System (SUS)—organized through a decentralized, participatory and equity‐oriented model that ensures universal, comprehensive and free healthcare—has been essential in enabling coordinated action across different levels of governance.

This experience demonstrates that elimination is feasible even in complex and heterogeneous settings when supported by strong governance, integrated health systems and community participation. This milestone reaffirms the country's commitment to sustaining these achievements while advancing towards the elimination of other vertically transmitted infections, including syphilis, hepatitis B, Chagas disease and HTLV, in alignment with global health agendas and the SDGs.

## Author Contributions

PCG has been involved in drafting the manuscript, interpretation of data, revising it critically for important intellectual content and has given final approval of the version to be published. AEM has been involved in drafting the manuscript, interpretation of data, revising it critically for important intellectual content and has given final approval of the version to be published. LHL has made substantial contributions to the acquisition, analysis and interpretation of data, and has given final approval of the version to be published. ARPP has made substantial contributions to the acquisition, analysis and interpretation of data, and has given final approval of the version to be published. CSBD has made substantial contributions to the acquisition, analysis and interpretation of data, and has given final approval of the version to be published. IVAD has made substantial contributions to the acquisition, analysis and interpretation of data, and has given final approval of the version to be published. AK has made substantial contributions to the acquisition, analysis and interpretation of data, and has given final approval of the version to be published. APBS has made substantial contributions to the acquisition, analysis and interpretation of data, and has given final approval of the version to be published. AB has been involved in drafting the manuscript and revising it critically for important intellectual content, and has given final approval of the version to be published. ATBM has been involved in drafting the manuscript and revising it critically for important intellectual content, and has given final approval of the version to be published. CGS has been involved in drafting the manuscript and revising it critically for important intellectual content, and has given final approval of the version to be published. AGM has been involved in revising it critically for important intellectual content and has given final approval of the version to be published. ASB has been involved in drafting the manuscript and revising it critically for important intellectual content, and has given final approval of the version to be published. DB has been involved in drafting the manuscript and revising it critically for important intellectual content, and has given final approval of the version to be published. MBGS has been involved in drafting the manuscript and revising it critically for important intellectual content, and has given final approval of the version to be published. BEVTG has been involved in drafting the manuscript and revising it critically for important intellectual content, and has given final approval of the version to be published.

## Funding

The study was funded by the Department of HIV, AIDS, Tuberculosis, Viral Hepatitis, and Sexually Transmitted Infections—Dathi, Secretariat for Health and Environmental Surveillance—SVSA, Ministry of Health of Brazil—MoH.

## Conflicts of Interest

The authors declare no conflicts of interest.

## Data Availability

This commentary did not generate or analyze original datasets. The referenced information is publicly available through the Validation Report for the Elimination of HIV Vertical Transmission in Brazil, available at https://www.gov.br/aids/pt-br/central-de-conteudo/publicacoes/2026/brazil_validation_report_hiv_emtct_ingles-final.pdf
